# Supplementary material for: The drug-induced phenotypic landscape of colorectal cancer organoids
Source: Nat Commun. 2022 Jun 6;13:3135. doi: 10.1038/s41467-022-30722-9 (PMC9170716; doi:10.1038/s41467-022-30722-9)
Supplement: Supplementary file 1 — Supplementary Information [file 41467_2022_30722_MOESM1_ESM.pdf]

Supplementary Information

The drug-induced phenotypic landscape of colorectal cancer organoids

Johannes Betge[*], Niklas Rindtorff[*], Jan Sauer[*], Benedikt Rauscher[*], Clara Dingert, Haristi Gaitantzi, Frank Herweck, Kauthar Srour-Mhanna, Thilo Miersch, Erica Valentini, Kim E. Boonekamp, Veronika Hauber, Tobias Gutting, Larissa Frank, Sebastian Belle, Timo Gaiser, Inga Buchholz, Ralf Jesenofsky, Nicolai Härtel, Tianzuo Zhan, Bernd Fischer, Katja Breitkopf-Heinlein, Elke Burgermeister, Matthias P. Ebert[#], Michael Boutros[#]

Supplemental Figures

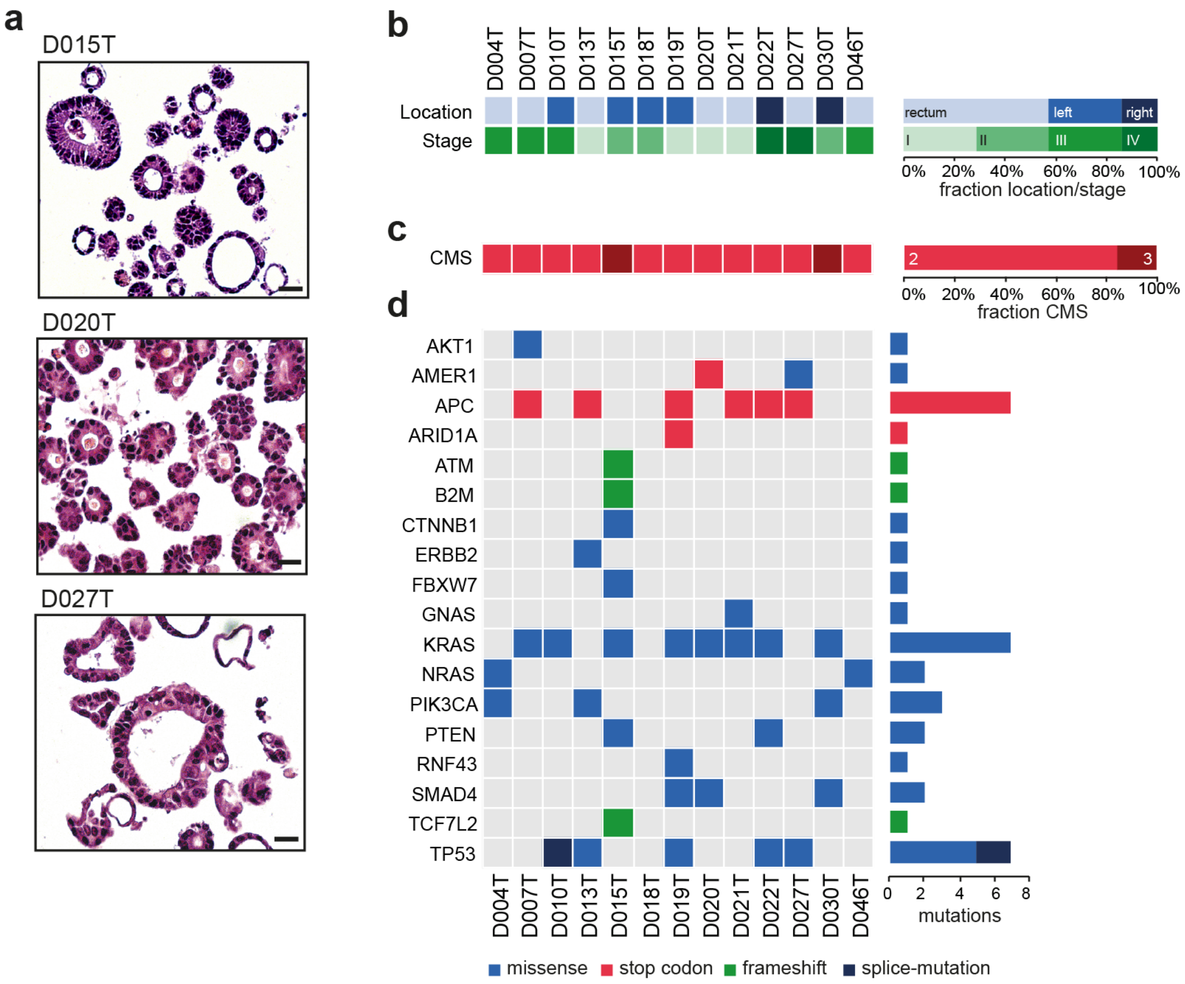

Supplemental Fig. S1: Establishment of patient derived organoids for high-throughput image-based compound profiling. **a**, Examples of H&E stained slides of selected representative PDO cultures; scale bar: 25µm. **b**, Tumor location (right/left/rectum) and AJCC/UICC stage of colorectal cancers that PDOs were derived from. **c**, Consensus molecular subtypes of PDOs determined by RNA expression analysis. **d**, Mutation status in PDOs, as analyzed by amplicon sequencing (more information in Supplemental Table S1).

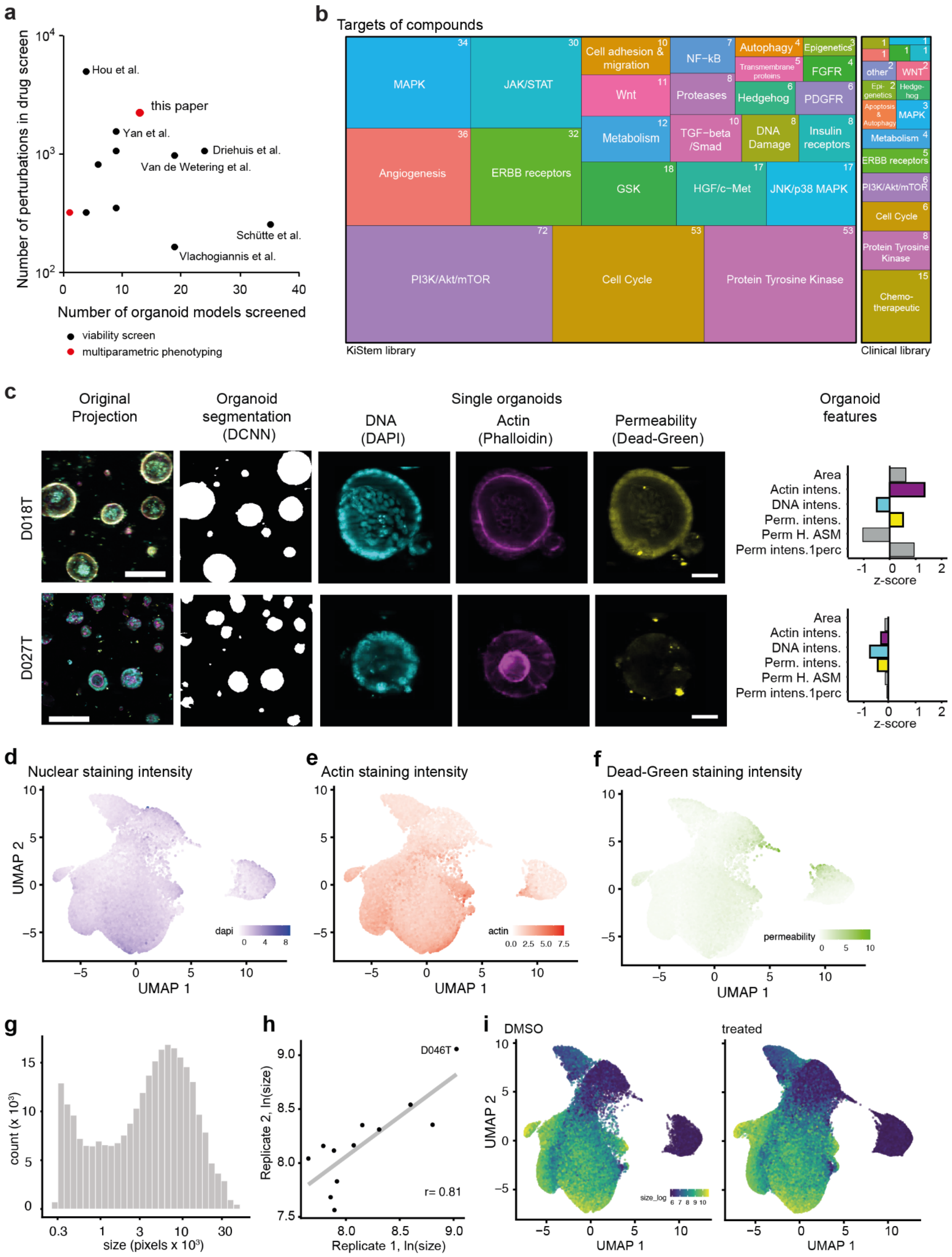
a
Number of perturbations in drug screen
Number of organoid models screened
Hou et al.
this paper
Yan et al.
Driehuis et al.
Van de Wetering et al.
Schütte et al.
Vlachogiannis et al.
viability screen
multiparametric phenotyping
b
Targets of compounds
MAPK
JAK/STAT
Cell adhesion & migration
NF-kB
Autophagy
Epigenetics
Transmembrane proteins
FGFR
Wnt
Proteases
Hedgehog
PDGFR
Metabolism
TGF-beta /Smad
DNA Damage
Insulin receptors
Angiogenesis
ERBB receptors
GSK
HGF/c-Met
JNK/p38 MAPK
PI3K/Akt/mTOR
Cell Cycle
Protein Tyrosine Kinase
other
WNT
Epi-genetics
Hedge-hog
Apoptosis & Autophagy
Chemo-therapeutic
KiStem library
Clinical library
c
Original Projection
Organoid segmentation (DCNN)
Single organoids
DNA (DAPI)
Actin (Phalloidin)
Permeability (Dead-Green)
Organoid features
D018T
D027T
Area
Actin intens.
DNA intens.
Perm. intens.
Perm H. ASM
Perm intens.1perc
z-score
d
Nuclear staining intensity
UMAP 2
UMAP 1
dapi
e
Actin staining intensity
actin
f
Dead-Green staining intensity
permeability
g
count (x 10³)
size (pixels x 10³)
h
Replicate 2, ln(size)
Replicate 1, ln(size)
D046T
r= 0.81
i
DMSO
treated
size_log

Supplemental Fig. S2: Automated morphological analysis of patient derived organoids. a, Number of organoid models and number of perturbations in previous publications reporting high-throughput drug screenings with patient derived cancer organoids **b**, Graphical representation of the compound libraries used for drug screening in this project: A library targeting kinases and stem cell pathways (KiStem library, 464 compounds) and a clinical library with 63 drugs in 5 concentrations. **c**, The image-processing pipeline illustrated with representative example images from 2 organoid lines: The multi-channel (DNA/DAPI, actin/TRITC, permeability/FITC) 3D image stack was projected onto a plane and a deep convolutional neural network subsequently recognized complete foreground organoids. Descriptive features were extracted from all three channels to quantify phenotypes. Feature plots show the median phenotype of unperturbed organoids, six example features (Area, Phalloidin intensity, DAPI intensity, FITC intensity, FITC Haralic angular second moment (ASM) and FITC intensity 1-percentile) and their z-scores relative to all profiled organoid lines are shown. Scale bars: 200µm (left images / projection and segmentation), 50µm (right images / single organoids). **d-f**, Uniform Manifold Approximation and Projection (UMAP) of organoid-level features marked by DNA (DAPI) staining intensity (d), actin (Phalloidi/FITC) staining intensity (e) and permeability (DeadGreen) staining intensity (f). **g**, Distribution of organoid size in control (DMSO) treated organoid lines. **h**, Replicate correlation of organoid size in control treated organoids. **i**, UMAP representation of DMSO treated and drug treated organoids.

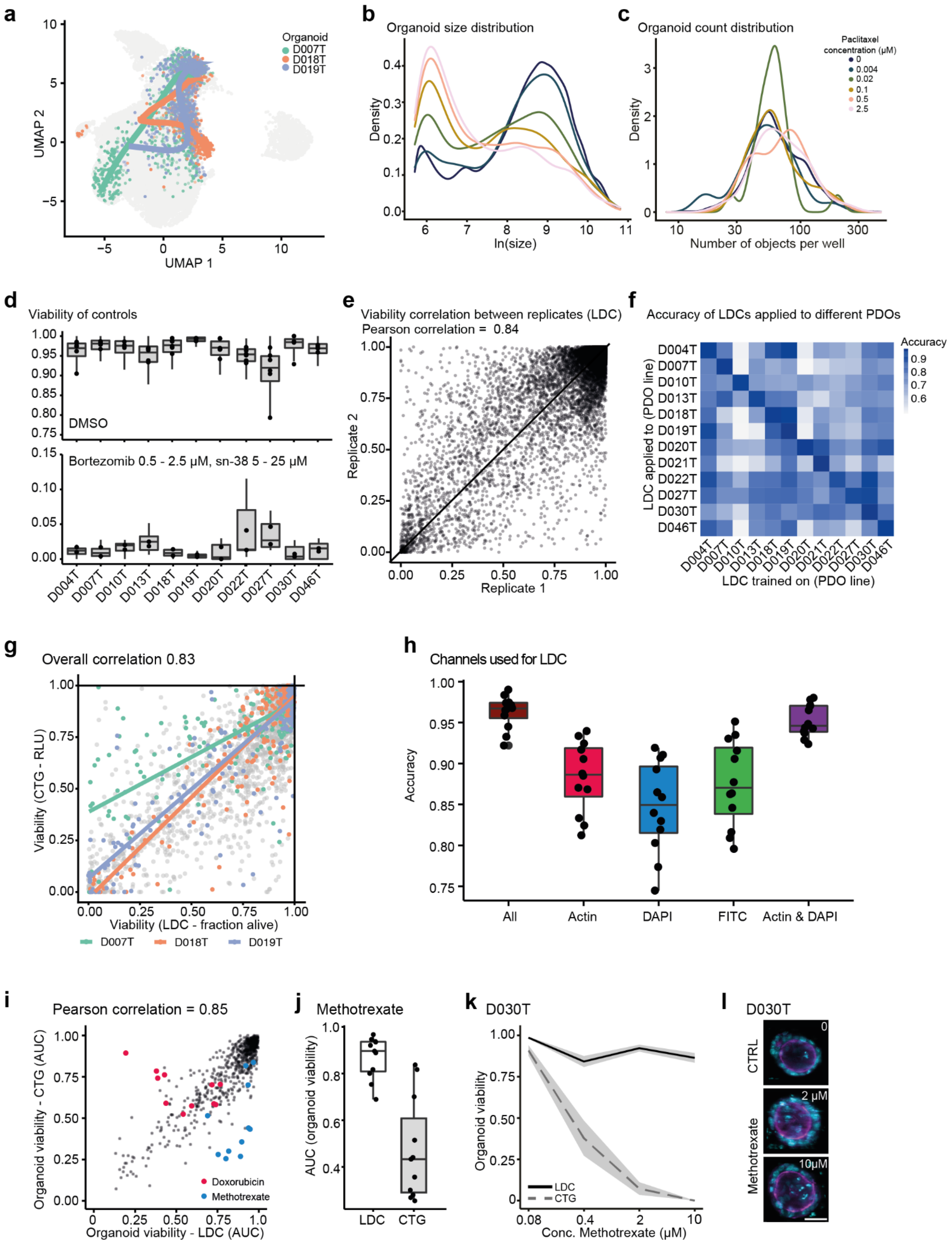

a
Organoid
D007T
D018T
D019T
UMAP 2
UMAP 1
b
Organoid size distribution
Density
ln(size)
c
Organoid count distribution
Paclitaxel concentration (μM)
Density
Number of objects per well
d
Viability of controls
DMSO
Bortezomib 0.5 - 2.5 μM, sn-38 5 - 25 μM
e
Viability correlation between replicates (LDC)
Pearson correlation = 0.84
Replicate 2
Replicate 1
f
Accuracy of LDCs applied to different PDOs
Accuracy
LDC applied to (PDO line)
LDC trained on (PDO line)
g
Overall correlation 0.83
Viability (CTG - RLU)
Viability (LDC - fraction alive)
h
Channels used for LDC
Accuracy
All
Actin
DAPI
FITC
Actin & DAPI
i
Pearson correlation = 0.85
Organoid viability - CTG (AUC)
Organoid viability - LDC (AUC)
Doxorubicin
Methotrexate
j
Methotrexate
AUC (organoid viability)
LDC
CTG
k
D030T
Organoid viability
Conc. Methotrexate (μM)
l
D030T
CTRL
Methotrexate

Supplemental Fig. S3: Analysis of lethal and non-lethal PDO phenotypes. a, Dose-dependent-trajectory of sn-38 drug effect. UMAP of organoid-level features are shown for example organoid lines D007T, D018T, D019T. For visual purposes, trajectory inference was limited to partition 1, the left-hand set of measurements within the UMAP representing ca. 95% of all imaging data. **b**, Distribution of organoid size at different concentrations of paclitaxel. Shown is a random sample of 30% of all paclitaxel treated organoids for this and the following figures. **c**, Distribution of organoid number per well at different concentrations of paclitaxel. **d-f**, Supervised machine learning of organoid viability. Organoid phenotype based viability classifiers (live-dead classifiers, LDC) were trained on positive- (high-dose bortezomib and SN-38) and negative (DMSO) controls. **d**, Plate-wise depiction of the fraction of DMSO treated (correctly) classified as viable (top) and fraction of organoids classified as dead in positive controls (bottom) for each PDO line. Data points average all organoids of DMSO treated wells (top) or bortezomib 0.5-2.5µM and sn-38 5-25µM treated wells per 384-well plate, respectively (i.e. 2 replicates of 2 plates KiStem library, 2 replicates of 2 plates clinical cancer library per line). Box indicates median, Q1 and Q3, whiskers indicate min and max. **e**, Correlation of viability (fraction of viable organoids per well) between 2 biological replicates classified by LDC. **f**, Transfer learning of organoid viability. LDCs were trained on the feature sets of negative- and positive controls for every PDO line. Receiver operator characteristic curves (ROCs) were analyzed on validation sets of negative- and positive controls of the same lines and other lines. Systematic analysis of the transfer-performance of all LDCs when applied across data from all organoid lines is shown. Classification performance is measured as the AUROC (area under the receiver operating characteristic curve). **g**, Association of organoid viability of selected example lines as determined by LDC vs. luminescence-based, ATP-dependent viability profiling with CellTiter-Glo (CTG). **h**, Accuracy of LDCs trained on image-features of all three available fluorescence channels compared to classifiers trained on single-channel data (actin/TRIC, DNA/DAPI, cell permeability/FITC) and on a combination of actin (TRIC) and DNA (DAPI) data only. All tested organoid lines were included in this analysis (2 replicates each), points depict accuracies of individual lines. Box indicates median, Q1 and Q3, whiskers indicate min and max. **i**, Pearson correlation of areas under the dose-response curve (AUCs) for drugs with multiple tested concentrations. Shown are AUCs for phenotype based viability classification (x-axis) and CTG ground truth (r = 0.87). Measurements of methotrexate and doxorubicin are marked in blue and red, respectively. All profiled lines are included. **j**, Methotrexate, an example of a drug that had divergent viability response results between phenotype based and CTG measurements. Data points represent AUCs calculated from dose-response curves generated with LDC or CTG measurement of 11 organoid lines (averages of 2 biological replicates). Box indicates median, Q1 and Q3, whiskers indicate min and max. **k**, Representative example of dose-response curves (organoid line D030T) for methotrexate. The range of min. - max. of LDC-values from two biological replicates are shaded in grey. **l**, Representative images of organoids treated with DMSO (control) and methotrexate 2 and 10 µM.; cyan = DAPI, magenta = Phalloidin, yellow = cell permeability; scale-bar: 50µm.

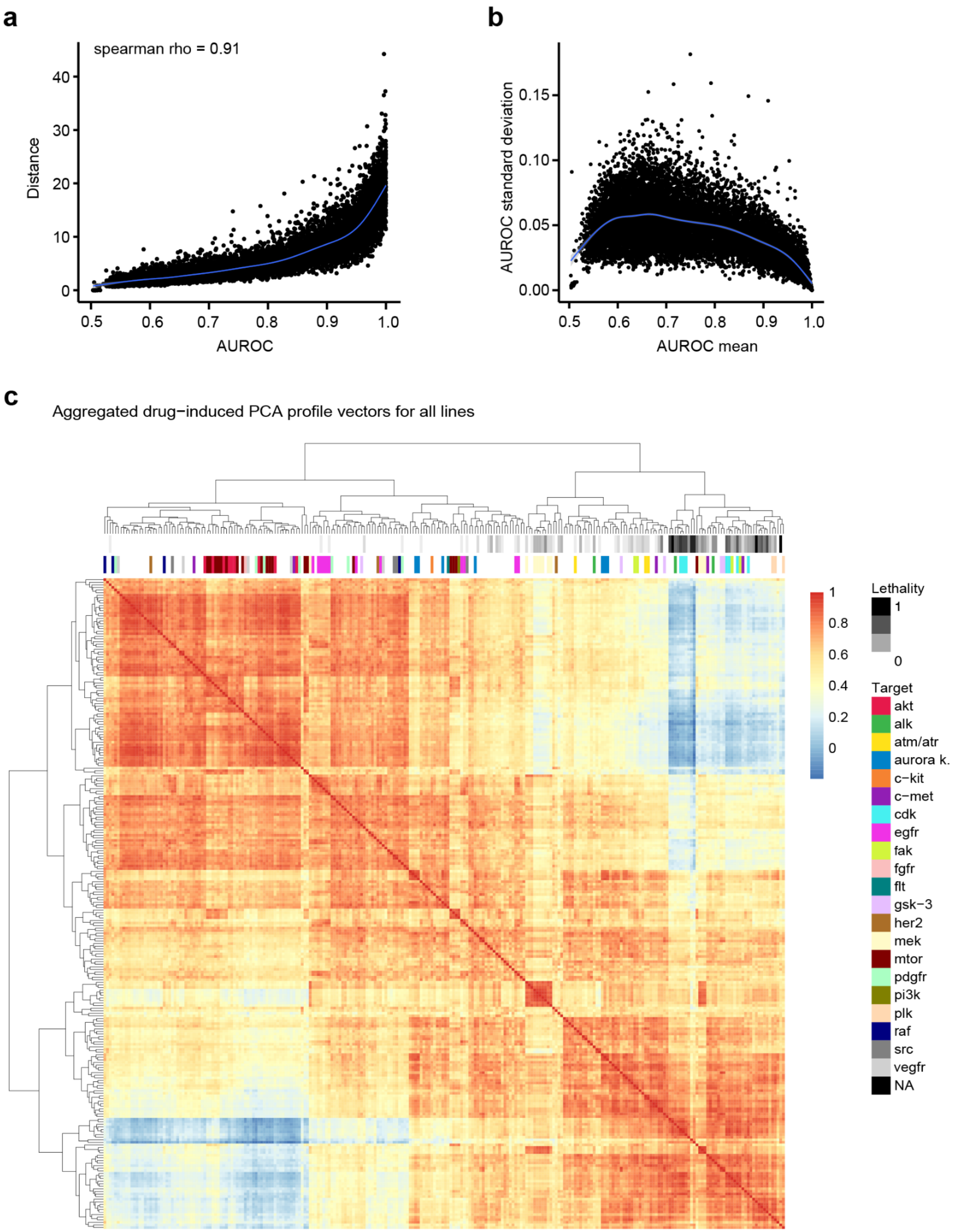
a
spearman rho = 0.91
Distance
AUROC
b
AUROC standard deviation
AUROC mean
c
Aggregated drug−induced PCA profile vectors for all lines
Lethality
1
0
Target
akt
alk
atm/atr
aurora k.
c−kit
c−met
cdk
egfr
fak
fgfr
flt
gsk−3
her2
mek
mtor
pdgfr
pi3k
plk
raf
src
vegfr
NA

Supplemental Fig. S4: Comparison of drug activity metrics. a, comparison of euclidean distance and classifier performance (AUROC) to describe drug activity. **b**, average AUROC observed and standard deviation of AUROC estimates after 10-fold cross validation. **c**. Hierarchical clustering of active compound effects by pearson correlation. Compound effects were determined by concatenating average morphology profiles from all organoid lines (25 principal components).

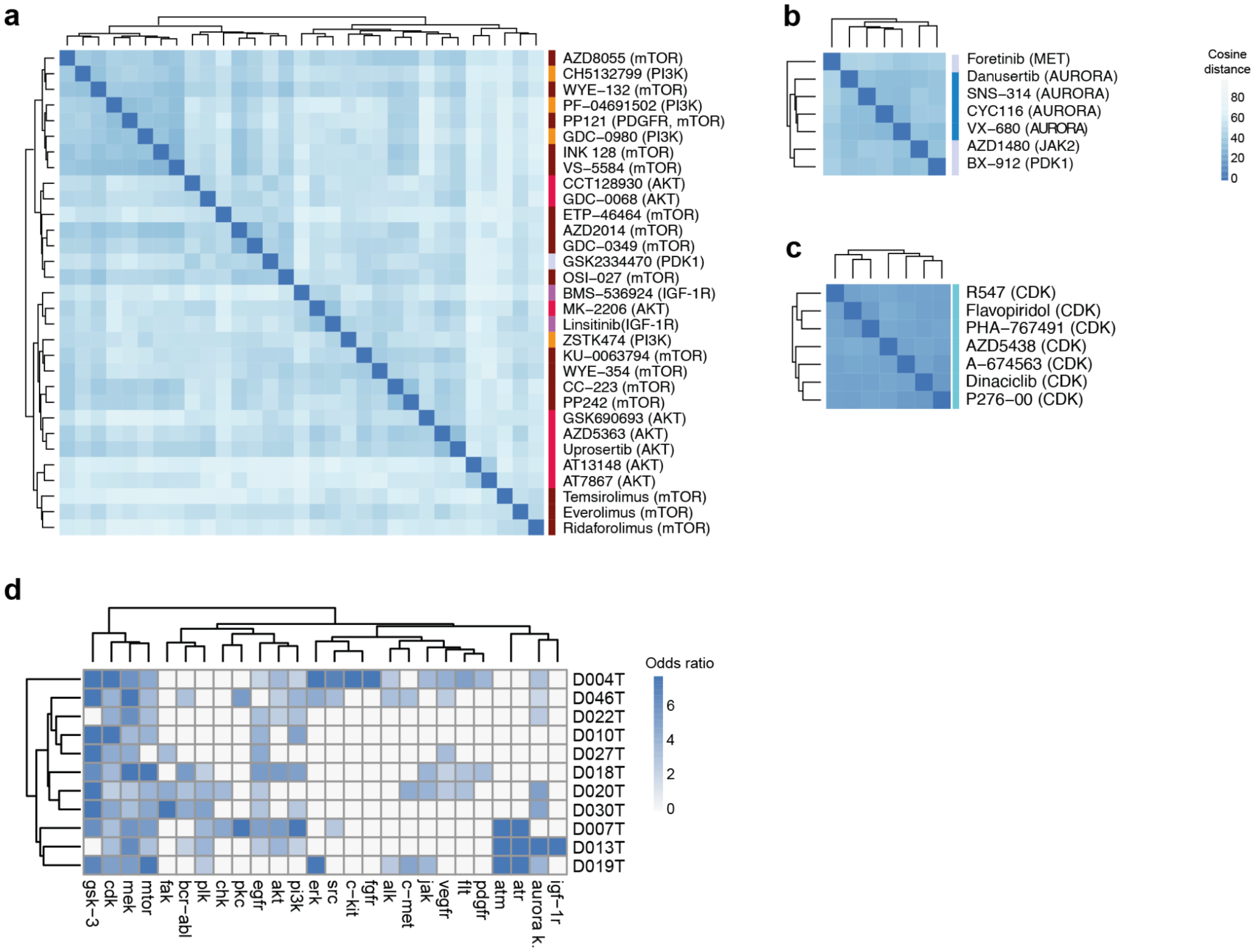

Supplemental Fig. S5: Selected drug-induced profile clusters (From Fig. 3d). **a-c**, shown are clusters enriched for PI3K/AKT/mTOR signaling inhibitors, Aurora kinase and CDK inhibitors, respectively. **d**, Significantly enriched targets across profiled PDOs. The cosine distance between drug effect vectors in individual organoid models was clustered, enrichment of annotated molecular targets was tested using Fisher's exact test. Log odds ratios for enriched targets and organoid lines are shown.

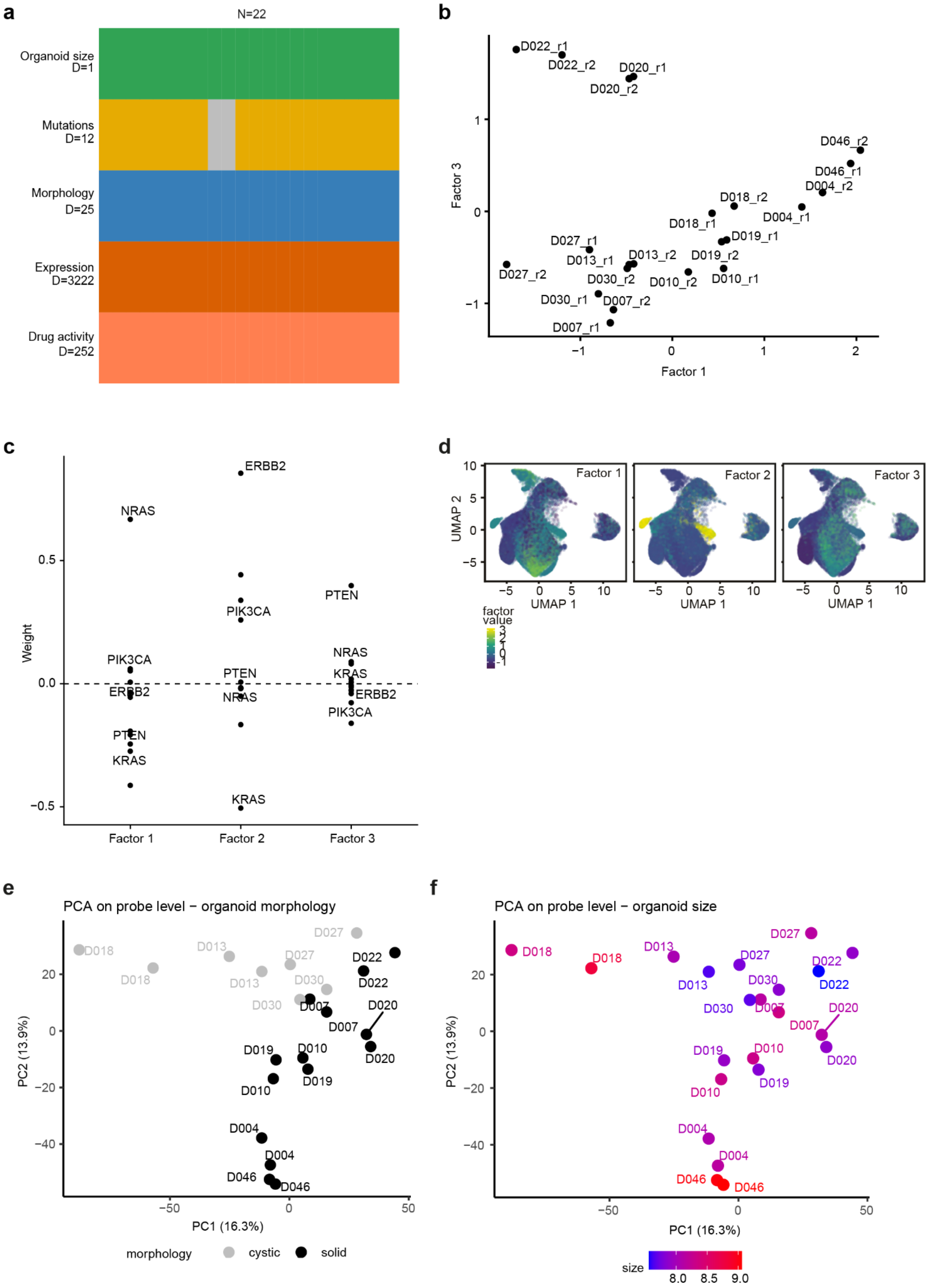
a
N=22
Organoid size D=1
Mutations D=12
Morphology D=25
Expression D=3222
Drug activity D=252
b
Factor 3
Factor 1
D022_r1
D022_r2
D020_r1
D020_r2
D046_r2
D046_r1
D004_r2
D018_r2
D004_r1
D018_r1
D019_r1
D027_r1
D013_r1
D013_r2
D019_r2
D027_r2
D030_r2
D010_r2
D010_r1
D030_r1
D007_r2
D007_r1
c
Weight
Factor 1
Factor 2
Factor 3
ERBB2
NRAS
PTEN
PIK3CA
KRAS
d
UMAP 2
UMAP 1
Factor 1
Factor 2
Factor 3
factor value
e
PCA on probe level − organoid morphology
PC2 (13.9%)
PC1 (16.3%)
morphology
cystic
solid
D018
D013
D027
D022
D030
D007
D020
D019
D010
D004
D046
f
PCA on probe level − organoid size
PC2 (13.9%)
PC1 (16.3%)
size
8.0
8.5
9.0

Supplemental Fig. S6: Multi-omics factor analysis overview. a, input data matrix showing the five included data modalities and 22 observations (11 lines a' 2 replicates). **b**, Factor 3 scores across unperturbed organoid lines. **c,** Factor loadings for somatic mutations, as identified by amplicon sequencing. **d**, UMAP embedding with organoid lines colored by factor scores. **e-f,** PCA analysis of expression data of unperturbed organoid lines. Two biological replicates were performed of all organoid lines. Colors represent organoid morphology (cystic vs. solid, visual inspection, e) and organoid size (f).

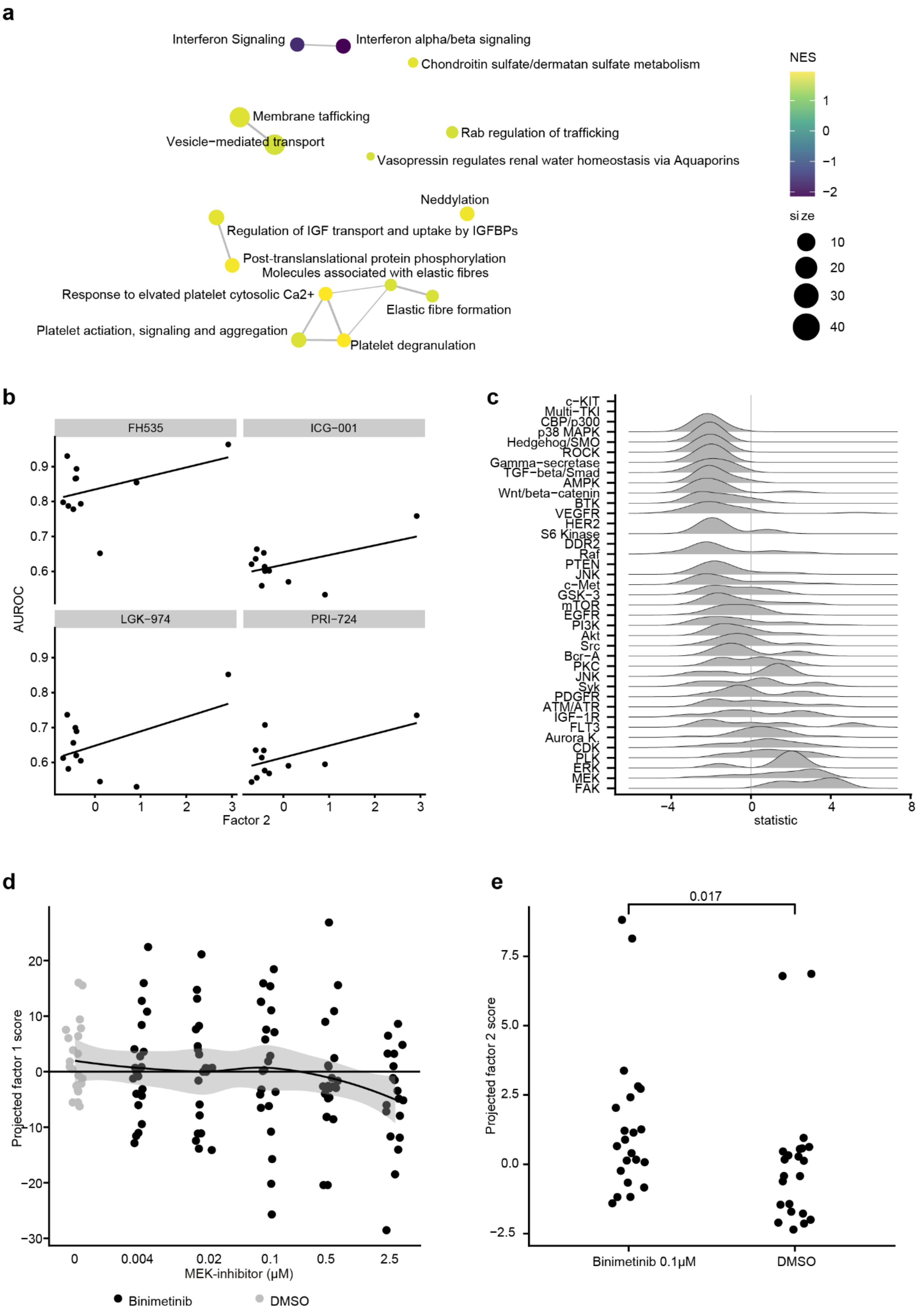
a
Interferon Signaling
Interferon alpha/beta signaling
Chondroitin sulfate/dermatan sulfate metabolism
Membrane tafficking
Vesicle−mediated transport
Rab regulation of trafficking
Vasopressin regulates renal water homeostasis via Aquaporins
Neddylation
Regulation of IGF transport and uptake by IGFBPs
Post-translanslational protein phosphorylation
Molecules associated with elastic fibres
Response to elvated platelet cytosolic Ca2+
Elastic fibre formation
Platelet actiation, signaling and aggregation
Platelet degranulation
NES
1
0
−1
−2
size
10
20
30
40
b
FH535
ICG−001
LGK−974
PRI−724
AUROC
0.9
0.8
0.7
0.6
0
1
2
3
Factor 2
c
c−KIT
Multi−TKI
CBP/p300
p38 MAPK
Hedgehog/SMO
ROCK
Gamma−secretase
TGF−beta/Smad
AMPK
Wnt/beta−catenin
BTK
VEGFR
HER2
S6 Kinase
DDR2
Raf
PTEN
JNK
c−Met
GSK−3
mTOR
EGFR
PI3K
Akt
Src
Bcr−A
PKC
JNK
Syk
PDGFR
ATM/ATR
IGF−1R
FLT3
Aurora K.
CDK
PLK
ERK
MEK
FAK
−4
0
4
8
statistic
d
Projected factor 1 score
20
10
0
−10
−20
−30
0
0.004
0.02
0.1
0.5
2.5
MEK-inhibitor (μM)
Binimetinib
DMSO
e
0.017
Projected factor 2 score
7.5
5.0
2.5
0.0
−2.5
Binimetinib 0.1μM
DMSO

Supplemental Fig. S7: Factor 2 defined by organoid LGR5 stem cell identity. a, Reactome pathway enrichment results for factor 2 gene expression loadings. Colorscale corresponds to normalized enrichment score (NES), where a positive value stands for an enrichment and a negative value for a depletion. Size of the nodes represent gene set size, edges correspond to gene set overlap **b,** Relationship of Wnt pathway inhibitor activity with factor 2 score. **c,** Approximated factor 2 shifts measured in organoids treated with small molecule inhibitors when accounting for line specific differences. Shifts are expressed as t statistics for the model (factor 2 ~ drug + line). **d,** Dose-dependent changes in factor 1 (IGFR1 signaling) scores after treatment with the MEK inhibitor binimitenib across organoid lines. The black line indicates median factor 2 values of 2 replicates from N=11 organoids treated with binimetinib, grey shading indicates the 95% confidence intervals based on the loess regression with default parameters. **e,** Factor 2 scores for organoids treated with 1µM binimetinib and DMSO control, data points shown represent the average factor scores of DMSO and binimetinib 0.1µM treated organoids from 11 lines (2 biological replicates / line) two-sided Wilcoxon rank sum test.

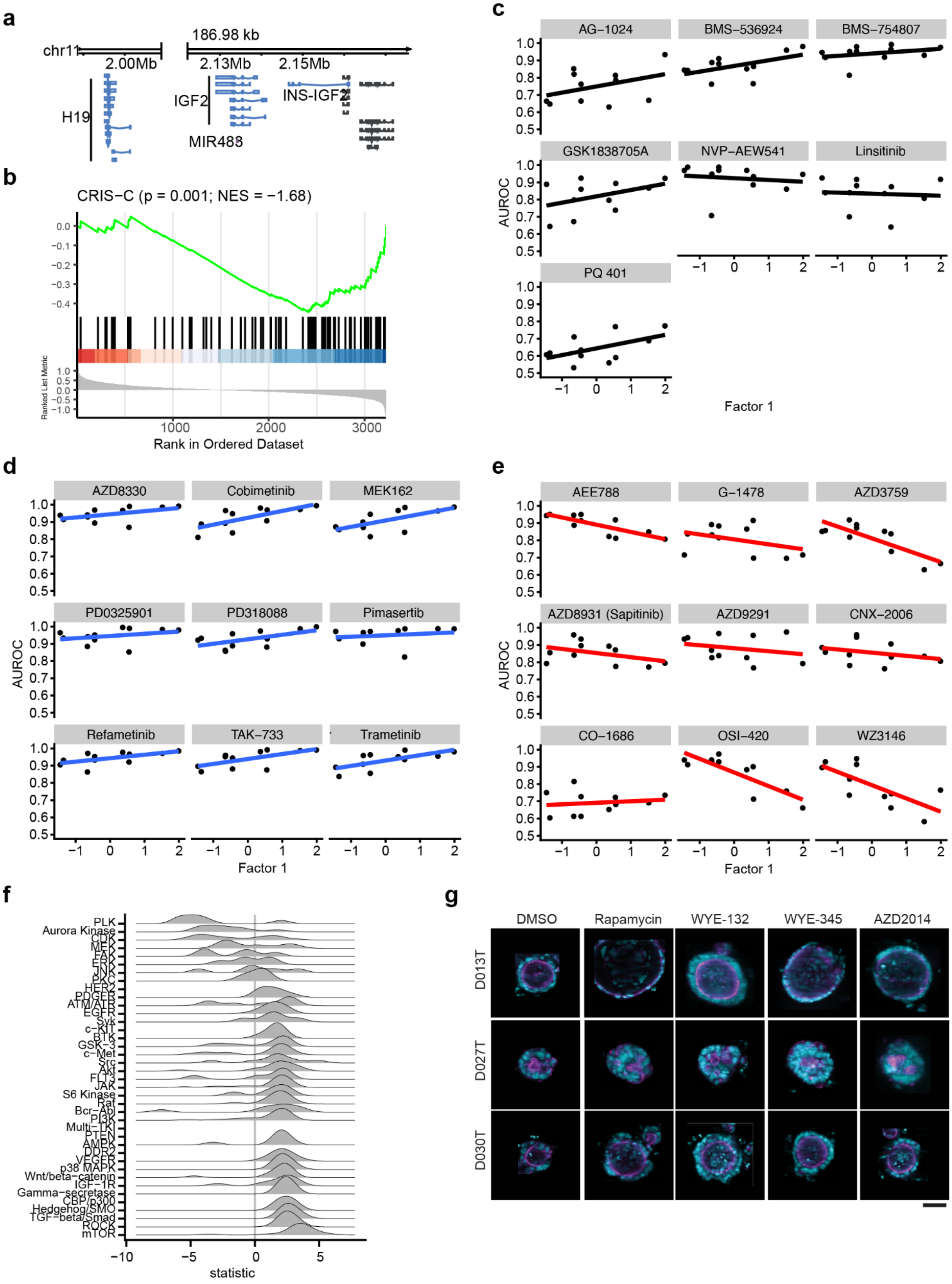
a
chr11
2.00Mb
186.98 kb
2.13Mb
2.15Mb
H19
IGF2
MIR483
INS-IGF2
b
CRIS−C (p = 0.001; NES = −1.68)
Ranked List Metric
Rank in Ordered Dataset
c
AG−1024
BMS−536924
BMS−754807
GSK1838705A
NVP−AEW541
Linsitinib
PQ 401
AUROC
Factor 1
d
AZD8330
Cobimetinib
MEK162
PD0325901
PD318088
Pimasertib
Refametinib
TAK−733
Trametinib
AUROC
Factor 1
e
AEE788
G−1478
AZD3759
AZD8931 (Sapitinib)
AZD9291
CNX−2006
CO−1686
OSI−420
WZ3146
AUROC
Factor 1
f
PLK
Aurora Kinase
CDK
MEK
FAK
ERK
JNK
PKC
HER
PDGFR
ATM/ATR
EGFR
Syk
c−Kit
BTK
GSK−3
c−Met
Src
Akt
FLT3
JAK
S6 Kinase
Raf
Bcr−Abl
PI3K
Multi−TKI
PTEN
AMPK
DDR2
VEGFR
p38 MAPK
Wnt/beta−catenin
IGF−1R
Gamma−secretase
CBP/p300
Hedgehog/SMO
TGF−beta/Smad
ROCK
mTOR
statistic
g
DMSO
Rapamycin
WYE−132
WYE−345
AZD2014
D013T
D027T
D030T

Supplemental Fig. S8: Factor 1 defined by IGF1R receptor signaling. a, Genomic track plot for *IGF2*, *H19* and *INS-IGF2*. **b**, gene set enrichment analysis for CRIS signature C on factor 1 gene expression loadings. **c-e,** Relationship of IGF1R-, MEK- and EGFR-inhibitor activity with factor 1 scores, respectively. Shown are 9 randomly sampled small molecule inhibitors. **f,** Approximated factor 1 shifts measured in organoids treated with small molecule inhibitors when accounting for line specific differences. Shifts are expressed as t statistics for the model (factor 1 ~ drug + line). **g**, Representative images of organois treated with DMSO (left) or selected mTOR inhibitors. Shown are images of organoid lines D013T, D027T and D030T (cyan = DNA, magenta = actin; sampled images were cropped and embedded in black background; scale bar: 50µm).

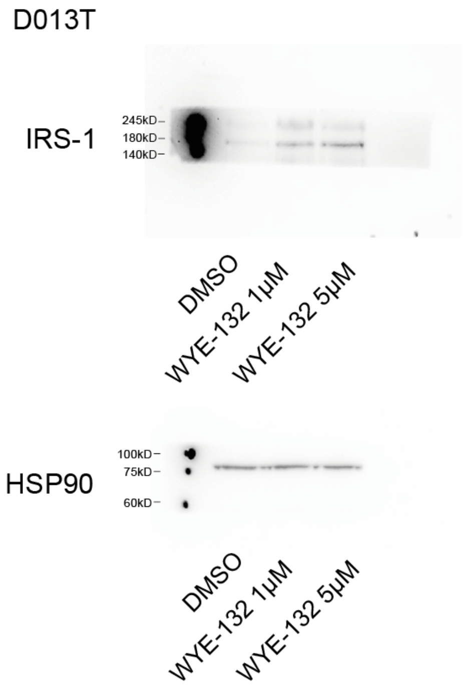

Supplemental Fig. S9: Complete scans of Western blot membranes in Fig. 6g

Supplemental Tables

Table S1: Patient and organoid characteristics

PDO line	PDO medium	PDO growth	Patient sex	Tumor location	Tumor stage (UICC)
**D004T**	ENA	good	f	rectum	3
**D007T**	ENA	good	m	rectum	3
**D010T**	ENA	good	m	sigm	3
**D013T**	ENAS	good	f	rectum	1
**D015T**	ENA	good	m	desc	2
**D018T**	ENA	good	m	sigm	2
**D019T**	ENA	good	f	sigm	1
**D020T**	ENA	good	m	rectum	1
**D021T**	ENA	medium	m	rectum	1
**D022T**	ENA	good	m	asc	4
**D027T**	ENA	good	m	rectum	4
**D030T**	ENA	good	f	asc	2
**D046T**	ENA	good	f	rectum	3

Abbreviations: f, female; m, male; sigm, sigmoid; desc, descending colon; asc, ascending colon; ENA, basal medium + EGF, Noggin and A83-01 (ALK-inhibitor); ENAS, ENA + SB202190 (p38 inhibitor).

Table S2: Mutations in organoids detected by amplicon sequencing

SAMPLE	SYMBOL	Protein_position	Amino_acids	Consequence
P004T	NRAS	61	Q/L	missense_variant
P004T	PIK3CA	546	Q/P	missense_variant
P007T	AKT1	17	E/K	missense_variant
P007T	APC	564	R/*	stop_gained
P007T	KRAS	12	G/D	missense_variant
P010T	KRAS	13	G/D	missense_variant
P010T	TP53	-	-	splice_acceptor_variant
P013T	APC	876	R/*	stop_gained
P013T	APC	1450	R/*	stop_gained
P013T	ERBB2	310	S/F	missense_variant
P013T	ERBB2	769	D/Y	missense_variant
P013T	PIK3CA	1004	M/I	missense_variant
P013T	TP53	273	R/C	missense_variant
P015T	ATM	2809	Q/X	frameshift_variant
P015T	B2M	14-15	SL/X	frameshift_variant
P015T	CTNNB1	34	G/V	missense_variant
P015T	FBXW7	465	R/C	missense_variant
P015T	KRAS	13	G/D	missense_variant
P015T	PTEN	160	T/I	missense_variant
P015T	TCF7L2	465	R/X	frameshift_variant
P019T	APC	1378	Q/*	stop_gained
P019T	ARID1A	1844	W/*	stop_gained
P019T	KRAS	146	A/T	missense_variant
P019T	RNF43	225	R/C	missense_variant
P019T	SMAD4	361	R/C	missense_variant
P019T	TP53	234	Y/N	missense_variant
P020T	AMER1	511	P/A	missense_variant
P020T	KRAS	12	G/V	missense_variant
P020T	SMAD4	102	P/R	missense_variant
P021T	APC	1356	S/*	stop_gained
P021T	GNAS	844	R/H	missense_variant
P021T	KRAS	12	G/D	missense_variant
P022T	APC	232	R/*	stop_gained
P022T	KRAS	12	G/C	missense_variant
P022T	PTEN	137	A/V	missense_variant
P022T	TP53	132	K/R	missense_variant
P027T	AMER1	373	A/G	missense_variant
P027T	APC	216	R/*	stop_gained
P027T	TP53	282	R/W	missense_variant
P030T	KRAS	146	A/T	missense_variant
P030T	PIK3CA	420	C/R	missense_variant
P030T	SMAD4	537	D/A	missense_variant
P046T	NRAS	61	Q/L	missense_variant

Table S3: Clinical cancer compound library

Compound Name	Target	Category	Phase	Max conc (µM)	Min conc (µM)
17AAG	HSP90	Targeted	Phase III	12,5	0,02
Afatinib	ErbB-Receptors	Targeted	Clinical use	100	0,16
Alisertib	Aurora A	Targeted	Phase II/III	25	0,04
Alpelisib	PIK3a	Targeted	Phase I	25	0,04
Axitinib	PDGFR, KIT, VEGFR	Targeted	Clinical use	75	0,12
AZD 4547	FGFR	Targeted	Phase II/III	25	0,04
AZD 5363	AKT	Targeted	Phase II	25	0,04
Bexaroten	Retinoid-Receptor	Targeted	Clinical use	20	0,032
Binimetinib	NRAS, MEK	Targeted	Phase III	25	0,04
Birinapant	SMAC	Targeted	Phase II	25	0,04
Bortezomib	Proteasom	Targeted	Clinical use	2,5	0,004
Cabozantinib	VEGF, MET, RET	Targeted	Clinical use	100	0,16
Crizotinib	ALK	Targeted	Clinical use	12,5	0,02
Dabrafenib	BRAF	Targeted	Clinical use	50	0,08
Dasatinib	DDR2, BCR-ABL, SRC, KIT, PDGFR	Targeted	Clinical use	100	0,16
Defactinib	FAK	Targeted	Phase II	7,5	0,012
Erlotinib	EGFR	Targeted	Clinical use	100	0,16
Everolimus	mTOR	Targeted	Clinical use	5	0,008
Gefitinib	EGFR	Targeted	Clinical use	100	0,16
GSK2636771	PTEN	Targeted	Phase I/II	25	0,04
Idelalisib	PIK3δ	Targeted	Clinical use	100	0,16
Lapatinib	HER2 and EGFR	Targeted	Clinical use	100	0,16
LGK974	Porcupine	Targeted	Phase I	100	0,16
MK-1775	Wee1	Targeted	Phase II	25	0,04
MK-8776	Chk1	Targeted	Phase II	7,5	0,012
Napabucasin	STAT3	Targeted	Phase III	25	0,04
Nutlin3a	p53	Targeted	Preclinical	100	0,16
Olaparib	PARP	Targeted	Clinical use	100	0,16
Palbociclib	CDK	Targeted	Clinical use	5	0,008
Panobinostat	HDAC	Targeted	Clinical use	2,5	0,004
Pazopanib	c-KIT, FGFR, PDGFR and VEGFR	Targeted	Clinical use	100	0,16
Ponatinib	BEGFR, PDGFR, FGFR, EPH recep	Targeted	Clinical use	25	0,04
PRI-724	CBP/p300	Targeted	Phase I-II	75	0,12
Regorafenib	VEGFR-1/2/3, TIE-2, KIT, RET, RAF	Targeted	Clinical use	100	0,16
Ruxolitinib	JAK1 and JAK2	Targeted	Clinical use	100	0,16
Sonidegib	SMO	Targeted	Clinical use	100	0,16
Sorafenib	PDGFR, KIT, VEGFR	Targeted	Clinical use	100	0,16
Sunitinib	Multi-TKI	Targeted	Clinical use	50	0,08
Taselisib	PI3K	Targeted	Phase III	25	0,04
Trametinib	Mek1/2	Targeted	Clinical use	2,5	0,004
Venetoclax	Bcl-2	Targeted	Clinical use	25	0,04
Vismodegib	SMO	Targeted	Clinical use	100	0,16
Volasertib	PLK1	Targeted	Phase II	25	0,04
Vorinostat	HDAC	Targeted	Clinical use	100	0,16
VX-702	p38 MAPK	Targeted	Phase II	25	0,04
YM155	Survivin	Tagreted	Phase II	5	0,008
5-FU	Antimetabolite	Chemotherapy	Clinical use	100	0,16
Bleomycin	DNA-Damage	Chemotherapy	Clinical use	50	0,08
Dacarbazine	Alkylating	Chemotherapy	Clinical use	12,5	0,02
Docetaxel	Mikrotubuli	Chemotherapy	Clinical use	2,5	0,004
Doxorubicin	Intercalating, mRNA synthesis	Chemotherapy	Clinical use	50	0,08
Etoposid	Topoisomerase	Chemotherapy	Clinical use	100	0,16
Fludarabine	Antimetabolite	Chemotherapy	Clinical use	12,5	0,02
Gemcitabine	DMA-Synthesis	Chemotherapy	Clinical use	12,5	0,02
Irinotecan / SN-38	Topoisomerase	Chemotherapy	Clinical use	25	0,04
Methotrexate	Antimetabolite	Chemotherapy	Clinical use	50	0,08
Mitomycin C	Alkylating	Chemotherapy	Clinical use	12,5	0,02
Oxaliplatin	Alkylating	Chemotherapy	Clinical use	75	0,12
Paclitaxel	Mikrotubuli	Chemotherapy	Clinical use	2,5	0,004
Trifluoridin/Tipiracil	Antimetabolite	Chemotherapy	Clinical use	55	0,088
Vinblastin	Mikrotubuli	Chemotherapy	Clinical use	2,5	0,004
Miglitol	Antidiabetic	Metabolism	Clinical use	12,5	0,02
Phenformin	Antidiabetic	Metabolism	Clinical use	100	0,16

Table S4: Kinase-Stemcell (Ki-Stem) compound library

Compound name	Target	Pathway	conc (uM)
1-Azakenpaullone	GSK-3	PI3K/Akt/mTOR	7,5
10058-F4	c-Myc	Cell Cycle	7,5
3-Methyladenine	PI3K	PI3K/Akt/mTOR	7,5
7,8-Dihydroxyflavone	Trk receptor	Protein Tyrosine Kinase	7,5
A-674563	Akt, CDK, PKA	PI3K/Akt/mTOR	7,5
A-769662	AMPK	PI3K/Akt/mTOR	7,5
A66	PI3K	PI3K/Akt/mTOR	7,5
AC480 (BMS-599626)	HER2	Neuronal Signaling	7,5
Acadesine	AMPK	PI3K/Akt/mTOR	7,5
AEE788 (NVP-AEE788)	EGFR, Flt, VEGFR, HER2	Protein Tyrosine Kinase	7,5
Afatinib (BIBW2992)	EGFR	Protein Tyrosine Kinase	7,5
AG-1024	IGF-1R	Protein Tyrosine Kinase	7,5
AG-1478 (Tyrphostin AG-1478)	EGFR	Protein Tyrosine Kinase	7,5
AG-18	EGFR	Protein Tyrosine Kinase	7,5
AG-490 (Tyrphostin B42)	JAK, EGFR	Others	7,5
Akti-1/2	Akt	PI3K/Akt/mTOR	7,5
Alisertib (MLN8237)	Aurora Kinase	Others	7,5
AMG 337	c-Met	Protein Tyrosine Kinase	7,5
AMG-458	c-Met	Protein Tyrosine Kinase	7,5
AMG-900	Aurora Kinase	Cell Cycle	7,5
AMG319	PI3K	PI3K/Akt/mTOR	7,5
Amuvatinib (MP-470)	c-Met, c-Kit, PDGFR, Flt, c-RET	Protein Tyrosine Kinase	7,5
ANA-12	Trk receptor	Protein Tyrosine Kinase	7,5
Anacardic Acid	Histone Acetyltransferase	Epigenetics	7,5
AP26113	ALK	Protein Tyrosine Kinase	7,5
Apatinib	VEGFR	Protein Tyrosine Kinase	7,5
AR-A014418	GSK-3	PI3K/Akt/mTOR	7,5
AR-A014418	GSK-3	PI3K/Akt/mTOR	7,5
AS-252424	PI3K	PI3K/Akt/mTOR	7,5
AS-604850	PI3K	PI3K/Akt/mTOR	7,5
Asiatic Acid	p38 MAPK	MAPK	7,5
ASP3026	ALK	Protein Tyrosine Kinase	7,5
AST-1306	EGFR	Protein Tyrosine Kinase	7,5
Astragaloside A	others	TGF-beta/Smad	7,5
AT13148	Akt	PI3K/Akt/mTOR	7,5
AT7519	CDK	Cell Cycle	7,5
AT7867	Akt, S6 kinase	PI3K/Akt/mTOR	7,5
AT9283	Bcr-Abl, JAK, Aurora Kinase	Others	7,5
Aurora A Inhibitor I	Aurora Kinase	Cell Cycle	7,5
Avagacestat (BMS-708163)	Gamma-secretase	Proteases	7,5
AVL-292	BTK	Angiogenesis	7,5
Axitinib	VEGFR, PDGFR, c-Kit	Protein Tyrosine Kinase	7,5
AZ 628	Raf	MAPK	7,5
AZ 960	JAK	JAK/STAT	7,5
AZ20	ATM/ATR	PI3K/Akt/mTOR	7,5
AZD1080	GSK-3	PI3K/Akt/mTOR	7,5
AZD1208	Pim	JAK/STAT	7,5
AZD1480	JAK	JAK/STAT	7,5
AZD1480	JAK	JAK/STAT	7,5
AZD2014	mTOR	PI3K/Akt/mTOR	7,5
AZD2858	GSK-3	PI3K/Akt/mTOR	7,5
AZD2932	PDGFR	Protein Tyrosine Kinase	7,5
AZD3463	ALK	Protein Tyrosine Kinase	7,5
AZD3759	EGFR	Protein Tyrosine Kinase	7,5
AZD4547	FGFR	Angiogenesis	7,5
AZD5363	Akt	PI3K/Akt/mTOR	7,5
AZD5438	CDK	Cell Cycle	7,5
AZD6482	PI3K	PI3K/Akt/mTOR	7,5
AZD6738	ATM/ATR	PI3K/Akt/mTOR	7,5
AZD7762	Chk	Cell Cycle	7,5
AZD8055	mTOR	PI3K/Akt/mTOR	7,5
AZD8330	MEK	MAPK	7,5
AZD8931 (Sapitinib)	EGFR, HER2	Protein Tyrosine Kinase	7,5
AZD9291	EGFR	Protein Tyrosine Kinase	7,5
Barasertib (AZD1152-HQPA)	Aurora Kinase	Others	7,5
Baricitinib (LY3009104, INCB028	JAK	Epigenetics	7,5
BAY 11-7082	I_B/IKK	NF-_B	7,5
Bay 11-7085	I_B/IKK	NF-_B	7,5
BGT226 (NVP-BGT226)	PI3K, mTOR	PI3K/Akt/mTOR	7,5
BI 2536	PLK	Others	7,5
BI-78D3	JNK	MAPK	7,5
BI-D1870	S6 Kinase	PI3K/Akt/mTOR	7,5
Bikinin	GSK-3	PI3K/Akt/mTOR	7,5
BIO	GSK-3	PI3K/Akt/mTOR	7,5
BIRB 796 (Doramapimod)	p38 MAPK	MAPK	7,5

BIX 02188	MEK	MAPK	7,5
BKM120 (NVP-BKM120, Buparli	PI3K	PI3K/Akt/mTOR	7,5
BLZ945	CSF-1R	Protein Tyrosine Kinase	7,5
BMS-265246	CDK	Cell Cycle	7,5
BMS-345541	I_B/IKK	NF-_B	7,5
BMS-536924	IGF-1R	Protein Tyrosine Kinase	7,5
BMS-582949	p38 MAPK	MAPK	7,5
BMS-754807	IGF-1R	Others	7,5
BMS-777607	c-Met	Protein Tyrosine Kinase	7,5
BMS-794833	c-Met, VEGFR	Protein Tyrosine Kinase	7,5
BMS-833923	Hedgehog/Smoothened	GPCR & G Protein	7,5
Bosutinib (SKI-606)	Src	Angiogenesis	7,5
Brivanib (BMS-540215)	VEGFR, FGFR	GPCR & G Protein	7,5
Brivanib Alaninate (BMS-582664	VEGFR, FGFR	Others	7,5
BS-181 HCl	CDK	Cell Cycle	7,5
Butein	EGFR	Protein Tyrosine Kinase	7,5
BX-795	PDK-1, IKK	PI3K/Akt/mTOR	7,5
BX-912	PDK-1	PI3K/Akt/mTOR	7,5
BYL719	PI3K	PI3K/Akt/mTOR	7,5
Cabozantinib (XL184, BMS-907	VEGFR, c-Met, Flt, Tie-2, c-Kit	Others	7,5
CAL-101 (Idelalisib, GS-1101)	PI3K	PI3K/Akt/mTOR	7,5
CAY10505	PI3K	PI3K/Akt/mTOR	7,5
CC-223	mTOR	PI3K/Akt/mTOR	7,5
CCT128930	Akt	PI3K/Akt/mTOR	7,5
Cediranib (AZD2171)	VEGFR, Flt	Protein Tyrosine Kinase	7,5
CEP-32496	Raf	MAPK	7,5
CEP-33779	JAK	JAK/STAT	7,5
CGI1746	BTK	Angiogenesis	7,5
CGK 733	ATM/ATR	DNA Damage	7,5
CH5132799	PI3K, mTOR	PI3K/Akt/mTOR	7,5
CHIR-124	Chk	Cell Cycle	7,5
CHIR-98014	GSK-3	PI3K/Akt/mTOR	7,5
CHIR-99021 (CT99021)	GSK-3	PI3K/Akt/mTOR	7,5
Chrysophanic Acid	EGFR, mTOR	Protein Tyrosine Kinase	7,5
CNX-2006	EGFR	Protein Tyrosine Kinase	7,5
CNX-774	BTK	Angiogenesis	7,5
CO-1686 (AVL-301)	EGFR	Protein Tyrosine Kinase	7,5
Cobimetinib (GDC-0973, RG742	MEK	MAPK	7,5
CP-673451	PDGFR	Protein Tyrosine Kinase	7,5
CP-724714	EGFR, HER2	Protein Tyrosine Kinase	7,5
Crenolanib (CP-868596)	PDGFR	Protein Tyrosine Kinase	7,5
Crizotinib (PF-02341066)	c-Met, ALK	Others	7,5
CUDC-101	HDAC, EGFR, HER2	Epigenetics	7,5
CUDC-907	HDAC, PI3K	Cytoskeletal Signaling	7,5
CX-6258 HCl	Pim	JAK/STAT	7,5
CYC116	Aurora Kinase, VEGFR	Cell Cycle	7,5
CYT387	JAK	JAK/STAT	7,5
CZC24832	PI3K_	PI3K/Akt/mTOR	7,5
D 4476	CK	Metabolism	7,5
Dabrafenib (GSK2118436)	Raf	MAPK	7,5
Dacomitinib (PF299804, PF299)	EGFR	Protein Tyrosine Kinase	7,5
Danusertib (PHA-739358)	Aurora Kinase, FGFR, Bcr-Abl,	Others	7,5
Daphnetin	PKA/PKC/EGFR	DNA Damage	7,5
DAPT (GSI-IX)	Gamma-secretase, Beta Amyloi	Proteases	7,5
DASA-58	PKM2	Others	7,5
Dasatinib	Src, Bcr-Abl, c-Kit	Angiogenesis	7,5
DCC-2036 (Rebastinib)	Bcr-Abl	Angiogenesis	7,5
DDR1-IN-1	DDR(receptor tyrosine kinase)	Others	7,5
Decernotinib (VX-509)	JAK	JAK/STAT	7,5
Degrasyn (WP1130)	DUB, Bcr-Abl	Angiogenesis	7,5
Dinaciclib (SCH727965)	CDK	Cell Cycle	7,5
Dovitinib (TKI-258, CHIR-258)	c-Kit, FGFR, Flt, VEGFR, PDGF	Angiogenesis	7,5
Dovitinib Dilactic Acid	Flt, FGFR, PDGFR, VEGFR, c-	Angiogenesis	7,5
EHop-016	Rac	Cell Cycle	7,5
ENMD-2076	Flt, Aurora Kinase, VEGFR	Angiogenesis	7,5
Entrectinib (RXDX-101)	Trk receptor	Protein Tyrosine Kinase	7,5
Enzastaurin (LY317615)	PKC	Neuronal Signaling	7,5
ERK5-IN-1	ERK	MAPK	7,5
ETC-1002	AMPK	PI3K/Akt/mTOR	7,5
ETP-46464	mTOR	PI3K/Akt/mTOR	7,5
Everolimus (RAD001)	mTOR	Others	7,5
Fasudil (HA-1077) HCl	ROCK	Cell Cycle	7,5
FH535	Wnt/beta-catenin	Stem Cells & Wnt	7,5
FIIN-2	FGFR	Protein Tyrosine Kinase	7,5
Filgotinib (GLPG0634)	JAK	JAK/STAT	7,5
Fingolimod (FTY720) HCl	S1P Receptor, Bcr-Abl, PKC	GPCR & G Protein	7,5
Flavopiridol (Alvocidib)	CDK	Cell Cycle	7,5
Foretinib (GSK1363089)	c-Met, VEGFR	Others	7,5

Fostamatinib (R788)	Syk	Angiogenesis	7,5
FRAX597	PAK	Cytoskeletal Signaling	7,5
G-749	FLT3	Angiogenesis	7,5
GDC-0068	Akt	PI3K/Akt/mTOR	7,5
GDC-0349	mTOR	PI3K/Akt/mTOR	7,5
GDC-0879	Raf	Others	7,5
GDC-0941	PI3K	Metabolism	7,5
GDC-0980 (RG7422)	mTOR, PI3K	PI3K/Akt/mTOR	7,5
Gefitinib (ZD1839)	EGFR	Protein Tyrosine Kinase	7,5
Genistein	EGFR	Protein Tyrosine Kinase	7,5
GF109203X	PKC	TGF-beta/Smad	7,5
GF109203X	PKC	TGF-beta/Smad	7,5
GNE-0877	leucine-rich repeat kinase 2 (LR	Autophagy	7,5
GNE-7915	LRRK	Autophagy	7,5
GNE-9605	LRRK2	Autophagy	7,5
GNF-2	Bcr-Abl	Angiogenesis	7,5
GNF-5	Bcr-Abl	Angiogenesis	7,5
Go 6983	PKC	TGF-beta/Smad	7,5
Golvatinib (E7050)	c-Met, VEGFR	Protein Tyrosine Kinase	7,5
GSK1838705A	IGF-1, ALK	Protein Tyrosine Kinase	7,5
GSK1904529A	IGF-1R	Others	7,5
GSK2126458 (GSK458)	PI3K, mTOR	PI3K/Akt/mTOR	7,5
GSK2292767	PI3K	PI3K/Akt/mTOR	7,5
GSK2334470	PDK-1	PI3K/Akt/mTOR	7,5
GSK2578215A	LRRK2	Autophagy	7,5
GSK2636771	PI3K	PI3K/Akt/mTOR	7,5
GSK429286A	ROCK	Cell Cycle	7,5
GSK461364	PLK	Cell Cycle	7,5
GSK621	AMPK	PI3K/Akt/mTOR	7,5
GSK650394	SGK	Others	7,5
GSK690693	Akt	Others	7,5
GW441756	Trk Receptor	Protein Tyrosine Kinase	7,5
GW5074	Raf	MAPK	7,5
GW788388	TGF-beta/Smad	TGF-beta/Smad	7,5
GZD824	Bcr-Abl	Angiogenesis	7,5
H 89 2HCl	S6 Kinase	PI3K/Akt/mTOR	7,5
Hesperadin	Aurora Kinase	Cell Cycle	7,5
Hesperetin	Histamine Receptor	TGF-beta/Smad	7,5
HMN-214	PLK	Cell Cycle	7,5
HO-3867	STAT	JAK/STAT	7,5
Honokiol	Akt, MEK	PI3K/Akt/mTOR	7,5
HS-173	PI3K	PI3K/Akt/mTOR	7,5
HTH-01-015	AMPK	PI3K/Akt/mTOR	7,5
Ibrutinib (PCI-32765)	Src	Angiogenesis	7,5
ICG-001	Wnt/beta-catenin	Stem Cells & Wnt	7,5
Icotinib	EGFR	Protein Tyrosine Kinase	7,5
IKK-16 (IKK Inhibitor VII)	IKK	NF-_B	7,5
IM-12	GSK-3	PI3K/Akt/mTOR	7,5
Imatinib Mesylate (STI571)	PDGFR, c-Kit, Bcr-Abl	Protein Tyrosine Kinase	7,5
IMD 0354	IKK	NF-_B	7,5
Indirubin	GSK-3	PI3K/Akt/mTOR	7,5
INK 128 (MLN0128)	mTOR	PI3K/Akt/mTOR	7,5
IPA-3	PAK	Cytoskeletal Signaling	7,5
IPI-145 (INK1197)	PI3K	Angiogenesis	7,5
IWP-L6	Wnt/beta-catenin	Stem Cells & Wnt	7,5
IWR-1-endo	Wnt/beta-catenin	Stem Cells & Wnt	7,5
JNJ-38877605	c-Met	Others	7,5
JNJ-7706621	CDK, Aurora Kinase	Cell Cycle	7,5
JNK Inhibitor IX	JNK	MAPK	7,5
JNK-IN-8	JNK	MAPK	7,5
K02288	TGF-beta/Smad	TGF-beta/Smad	7,5
Ki8751	VEGFR, c-Kit, PDGFR	Protein Tyrosine Kinase	7,5
KN-62	Ca2+/calmodulin-dependent pro	Others	7,5
KN-93 Phosphate	CaMK	Others	7,5
KRN 633	VEGFR, PDGFR	Protein Tyrosine Kinase	7,5
KU-0063794	mTOR	PI3K/Akt/mTOR	7,5
KU-55933 (ATM Kinase Inhibitor	ATM	Others	7,5
KU-60019	ATM	DNA Damage	7,5
KW-2449	Flt, Bcr-Abl, Aurora Kinase	Angiogenesis	7,5
KX2-391	Src	Angiogenesis	7,5
KY02111	Wnt/beta-catenin	Stem Cells & Wnt	7,5
Lapatinib	EGFR, HER2	Protein Tyrosine Kinase	7,5
Lapatinib (GW-572016) Ditosyla	EGFR, HER2	Protein Tyrosine Kinase	7,5
LDC000067	CDK	Cell Cycle	7,5
LDE225 (NVP-LDE225,Erismod	Smoothened	Stem Cells & Wnt	7,5
LDK378	ALK	Protein Tyrosine Kinase	7,5
LDN-214117	TGF-beta/Smad	TGF-beta/Smad	7,5
Lenvatinib (E7080)	VEGFR	Protein Tyrosine Kinase	7,5

LFM-A13	BTK	Angiogenesis	7,5
LGK-974	Wnt/beta-catenin	Stem Cells & Wnt	7,5
Linifanib (ABT-869)	PDGFR, VEGFR	Protein Tyrosine Kinase	7,5
LJH685	S6 Kinase	PI3K/Akt/mTOR	7,5
LJI308	S6 Kinase	PI3K/Akt/mTOR	7,5
Losmapimod (GW856553X)	p38 MAPK	MAPK	7,5
LY2090314	GSK-3	PI3K/Akt/mTOR	7,5
LY2157299	TGF-beta/Smad	TGF-beta/Smad	7,5
LY2603618	Chk	Cell Cycle	7,5
LY2784544	JAK	JAK/STAT	7,5
LY2784544	JAK	JAK/STAT	7,5
LY2811376	5-alpha Reductase	Proteases	7,5
LY2835219	CDK	Cell Cycle	7,5
LY294002	PI3K	Others	7,5
LY3023414	Akt	PI3K/Akt/mTOR	7,5
LY411575	Gamma-secretase	Proteases	7,5
Masitinib (AB1010)	c-Kit, PDGFR, FGFR, FAK	Others	7,5
MEK162 (ARRY-162, ARRY-43	MEK	MAPK	7,5
MGCD-265	c-Met, VEGFR, Tie-2	Protein Tyrosine Kinase	7,5
Milciclib (PHA-848125)	CDK	Cell Cycle	7,5
MK-0752	Gamma-secretase	Proteases	7,5
MK-2206 2HCl	Akt	Others	7,5
MK-2461	c-Met, FGFR, PDGFR	Protein Tyrosine Kinase	7,5
MK-5108 (VX-689)	Aurora Kinase	Cell Cycle	7,5
MK-8745	Aurora Kinase	Cell Cycle	7,5
MLN2480	Raf	MAPK	7,5
MLN8054	Aurora Kinase	Others	7,5
Motesanib Diphosphate (AMG-7	VEGFR, PDGFR, c-Kit	Protein Tyrosine Kinase	7,5
Mubritinib (TAK 165)	HER2	Protein Tyrosine Kinase	7,5
Nilotinib (AMN-107)	Bcr-Abl	Angiogenesis	7,5
Nintedanib (BIBF 1120)_uncerta	VEGFR, PDGFR, FGFR	Protein Tyrosine Kinase	7,5
NSC 23766	Rac	Cell Cycle	7,5
NU6027	CDK	Cell Cycle	7,5
NVP-AEW541	IGF-1R	Protein Tyrosine Kinase	7,5
NVP-BHG712	VEGFR, Src, Raf, Bcr-Abl	Protein Tyrosine Kinase	7,5
NVP-BSK805 2HCl	JAK	JAK/STAT	7,5
NVP-BVU972	c-Met	Protein Tyrosine Kinase	7,5
Oclacitinib	JAK	JAK/STAT	7,5
Olmutinib (HM61713, BI 148269	EGFR	Protein Tyrosine Kinase	7,5
ONO-4059	BTK	Angiogenesis	7,5
OSI-027	mTOR	PI3K/Akt/mTOR	7,5
OSI-420	EGFR	Protein Tyrosine Kinase	7,5
OSI-906 (Linsitinib)	IGF-1R	Others	7,5
OSI-930	c-Kit, VEGFR	Protein Tyrosine Kinase	7,5
OSU-03012 (AR-12)	PDK-1	Others	7,5
P276-00	CDK	Cell Cycle	7,5
Pacritinib (SB1518)	JAK	JAK/STAT	7,5
Palomid 529 (P529)	mTOR	PI3K/Akt/mTOR	7,5
Pazopanib	VEGFR	Protein Tyrosine Kinase	7,5
Pazopanib HCl	VEGFR, PDGFR, c-Kit	Protein Tyrosine Kinase	7,5
PD0325901	MEK	DNA Damage	7,5
PD168393	EGFR	Protein Tyrosine Kinase	7,5
PD173074	FGFR, VEGFR	Angiogenesis	7,5
PD173955	Bcr-Abl	Angiogenesis	7,5
PD184352 (CI-1040)	MEK	MAPK	7,5
PD318088	MEK	MAPK	7,5
PD98059	MEK	MAPK	7,5
Pelitinib (EKB-569)	EGFR	Protein Tyrosine Kinase	7,5
PF-00562271	FAK	Angiogenesis	7,5
PF-04217903	c-Met	Others	7,5
PF-04691502	mTOR, PI3K, Akt	PI3K/Akt/mTOR	7,5
PF-3758309	PAK	Cytoskeletal Signaling	7,5
PF-431396	FAK	Angiogenesis	7,5
PF-4708671	S6 Kinase	PI3K/Akt/mTOR	7,5
PF-477736	Chk	Cell Cycle	7,5
PF-5274857	Hedgehog/Smoothened	Stem Cells & Wnt	7,5
PF-543	SphK1	GPCR & G Protein	7,5
PF-562271	FAK	Angiogenesis	7,5
PF-573228	FAK	Angiogenesis	7,5
PFK15	6-phosphofructo-2-kinase (PFK	Others	7,5
PH-797804	p38 MAPK	MAPK	7,5
PHA-665752	c-Met	Others	7,5
PHA-680632	Aurora Kinase	Cell Cycle	7,5
PHA-767491	CDK	Cell Cycle	7,5
PHA-793887	CDK	Cell Cycle	7,5
Phenformin HCl	AMPK	PI3K/Akt/mTOR	7,5
PHT-427	Akt, PDK-1	PI3K/Akt/mTOR	7,5
PI-103	DNA-PK, PI3K, mTOR	Neuronal Signaling	7,5

Piceatannol	Syk	Angiogenesis	7,5
PIK-293	PI3K	PI3K/Akt/mTOR	7,5
PIK-294	PI3K	PI3K/Akt/mTOR	7,5
PIK-93	PI3K, VEGFR	PI3K/Akt/mTOR	7,5
Pimasertib (AS-703026)	MEK	MAPK	7,5
Pirfenidone	TGF-beta/Smad	TGF-beta/Smad	7,5
PLX-4720	Raf	Others	7,5
PLX7904	Raf	MAPK	7,5
Ponatinib (AP24534)	Bcr-Abl, VEGFR, FGFR, PDGF	Angiogenesis	7,5
PP1	Src	Angiogenesis	7,5
PP121	DNA-PK, mTOR, PDGF	Protein Tyrosine Kinase	7,5
PP2	Src	Angiogenesis	7,5
PP242	mTOR	PI3K/Akt/mTOR	7,5
PQ 401	IGF-1R	Protein Tyrosine Kinase	7,5
PRI-724	Wnt/beta-catenin	Stem Cells & Wnt	7,5
PRT062607 (P505-15, BIIB057)	Syk	Angiogenesis	7,5
Purvalanol A	CDK	Cell Cycle	7,5
Quercetin	PI3K, PKC, Src, Sirtuin	Epigenetics	7,5
Quizartinib (AC220)	Flt	Angiogenesis	7,5
R406	Syk, Flt	Angiogenesis	7,5
R406 (free base)	Syk	Angiogenesis	7,5
R547	CDK	Cell Cycle	7,5
RAF265 (CHIR-265)	Raf, VEGFR	MAPK	7,5
Rapamycin (Sirolimus)	mTOR	DNA Damage	7,5
Refametinib (RDEA119, Bay 86-	MEK	Others	7,5
Regorafenib (BAY 73-4506)	c-Kit, Raf, VEGFR	Protein Tyrosine Kinase	7,5
RepSox	TGF-beta/Smad	TGF-beta/Smad	7,5
Ridaforolimus (Deforolimus, MK-	mTOR	PI3K/Akt/mTOR	7,5
Rigosertib (ON-01910)	PLK	Cell Cycle	7,5
RKI-1447	ROCK	Cell Cycle	7,5
RN486	BTK	Angiogenesis	7,5
Ro 31-8220 Mesylate	PKC	TGF-beta/Smad	7,5
Ro-3306	CDK	Cell Cycle	7,5
Ro3280	PLK	Cell Cycle	7,5
RO4929097	Y-Secretase	Proteases	7,5
Roscovitine (Seliciclib,CYC202)	CDK	Others	7,5
Ruxolitinib (INCB018424)	JAK	JAK/STAT	7,5
S-Ruxolitinib (INCB018424)	JAK	JAK/STAT	7,5
SANT-1	Smoothened	Stem Cells & Wnt	7,5
SAR131675	VEGFR	Protein Tyrosine Kinase	7,5
SAR245409 (XL765)	PI3K, mTOR	PI3K/Akt/mTOR	7,5
Saracatinib (AZD0530)	Src, Bcr-Abl	Angiogenesis	7,5
SB202190 (FHPI)	p38 MAPK	PI3K/Akt/mTOR	7,5
SB203580	p38 MAPK	Transmembrane Transpc	7,5
SB216763	GSK-3	Others	7,5
SB239063	p38 MAPK	MAPK	7,5
SB415286	GSK-3	PI3K/Akt/mTOR	7,5
SB431542	TGF-beta/Smad	Others	7,5
SB505124	TGF-beta/Smad	TGF-beta/Smad	7,5
SB525334	TGF-beta/Smad	TGF-beta/Smad	7,5
SB590885	Raf	MAPK	7,5
SC-514	I_B/IKK	NF-_B	7,5
SC1	ERK	MAPK	7,5
Schisandrin B (Sch B)	ATM/ATR	PI3K/Akt/mTOR	7,5
Selumetinib (AZD6244)	MEK	MAPK	7,5
Semagacestat (LY450139)	Gamma-secretase	Proteases	7,5
Semaxanib (SU5416)	VEGFR	Protein Tyrosine Kinase	7,5
SGI-1776 free base	Pim	JAK/STAT	7,5
SGI-7079	VEGFR	Protein Tyrosine Kinase	7,5
SH-4-54	STAT	JAK/STAT	7,5
Skepinone-L	p38 MAPK	MAPK	7,5
SKI II	sphingosine kinase (SphK)	GPCR & G Protein	7,5
SL-327	MEK	Others	7,5
SMI-4a	Pim	JAK/STAT	7,5
SNS-032 (BMS-387032)	CDK	Others	7,5
SNS-314 Mesylate	Aurora Kinase	Others	7,5
Sorafenib	Raf	MAPK	7,5
Sotrastaurin	PKC	TGF-beta/Smad	7,5
SP600125	JNK	MAPK	7,5
SSR128129E	FGFR	Angiogenesis	7,5
STA-21	STAT	JAK/STAT	7,5
SU11274	c-Met	Neuronal Signaling	7,5
SU6656	Src	Angiogenesis	7,5
SU9516	CDK	Cell Cycle	7,5
Sunitinib Malate	VEGFR, PDGFR, c-Kit, Flt	Microbiology	7,5
TAE226 (NVP-TAE226)	FAK	Angiogenesis	7,5
TAK-285	EGFR, HER2	Protein Tyrosine Kinase	7,5
TAK-632	Raf	MAPK	7,5

TAK-715	p38 MAPK	MAPK	7,5
TAK-733	MEK	MAPK	7,5
TAK-901	Aurora Kinase	Cell Cycle	7,5
Taladegib (LY2940680)	Hedgehog,Hedgehog/Smoothen	Stem Cells & Wnt	7,5
TCS 359	FLT3	Angiogenesis	7,5
TDZD-8	GSK-3	PI3K/Akt/mTOR	7,5
Telatinib	VEGFR, PDGFR, c-Kit	Protein Tyrosine Kinase	7,5
Temsirolimus (CCI-779, NSC 6	mTOR	Neuronal Signaling	7,5
Tepotinib (EMD 1214063)	c-Met	Protein Tyrosine Kinase	7,5
TG003	CDK	Cell Cycle	7,5
TG100-115	PI3K	PI3K/Akt/mTOR	7,5
TG101209	Flt, JAK, c-RET	JAK/STAT	7,5
TG101348 (SAR302503)	JAK	JAK/STAT	7,5
TGX-221	PI3K	PI3K/Akt/mTOR	7,5
Theophylline	TGF-beta/Smad	TGF-beta/Smad	7,5
Thiazovivin	ROCK	Cell Cycle	7,5
TIC10 Analogue	Akt	PI3K/Akt/mTOR	7,5
Tideglusib	GSK-3	PI3K/Akt/mTOR	7,5
Tie2 kinase inhibitor	Tie-2	Protein Tyrosine Kinase	7,5
Tivantinib (ARQ 197)	c-Met	Protein Tyrosine Kinase	7,5
Tivozanib (AV-951)	VEGFR, c-Kit, PDGFR	Protein Tyrosine Kinase	7,5
Tofacitinib (CP-690550,Tasociti	JAK	JAK/STAT	7,5
Tofacitinib (CP-690550) Citrate	JAK	JAK/STAT	7,5
Torin 2	mTOR	PI3K/Akt/mTOR	7,5
TPCA-1	IKK	NF-_B	7,5
Trametinib (GSK1120212)	MEK	MAPK	7,5
Triciribine	Akt	Others	7,5
TSU-68 (SU6668, Orantinib)	VEGFR, PDGFR , FGFR	Protein Tyrosine Kinase	7,5
TWS119	GSK-3	PI3K/Akt/mTOR	7,5
TWS119	GSK-3	PI3K/Akt/mTOR	7,5
Tyrphostin 9	EGFR	Protein Tyrosine Kinase	7,5
Tyrphostin AG 1296	PDGFR	Protein Tyrosine Kinase	7,5
Tyrphostin AG 879	HER2	Protein Tyrosine Kinase	7,5
U0126-EtOH	MEK	Others	7,5
Ulixertinib (BVD-523, VRT75227	ERK	MAPK	7,5
Uprosertib (GSK2141795)	Akt	PI3K/Akt/mTOR	7,5
URMC-099	Abl1; LRRK2; MLK3; MLK1	Others	7,5
Vacquinol-1	JNK	MAPK	7,5
Varlitinib	EGFR	Protein Tyrosine Kinase	7,5
Vatalanib (PTK787) 2HCl	VEGFR, c-Kit, Flt	Others	7,5
VE-821	ATM/ATR	DNA Damage	7,5
VE-822	ATM/ATR	PI3K/Akt/mTOR	7,5
Vemurafenib (PLX4032, RG720	Raf	MAPK	7,5
Vismodegib (GDC-0449)	Hedgehog, P-gp	Neuronal Signaling	7,5
Volasertib (BI 6727)	PLK	Cell Cycle	7,5
VPS34-IN1	PI3K	PI3K/Akt/mTOR	7,5
VS-5584 (SB2343)	PI3K	PI3K/Akt/mTOR	7,5
VX-11e	ERK	MAPK	7,5
VX-680 (Tozasertib, MK-0457)	Aurora Kinase	Endocrinology & Hormon	7,5
VX-702	p38 MAPK	MAPK	7,5
VX-745	p38 MAPK	MAPK	7,5
WAY-600	mTOR	PI3K/Akt/mTOR	7,5
WH-4-023	Src	Angiogenesis	7,5
WHI-P154	JAK, EGFR	JAK/STAT	7,5
WIKI4	Wnt/beta-catenin	Stem Cells & Wnt	7,5
Wnt agonist 1	Wnt/beta-catenin	Stem Cells & Wnt	7,5
Wnt-C59 (C59)	Wnt/beta-catenin	Stem Cells & Wnt	7,5
WP1066	JAK	JAK/STAT	7,5
WYE-125132 (WYE-132)	mTOR	PI3K/Akt/mTOR	7,5
WYE-354	mTOR	PI3K/Akt/mTOR	7,5
WZ3146	EGFR	Protein Tyrosine Kinase	7,5
WZ4002	EGFR	Protein Tyrosine Kinase	7,5
WZ4003	AMPK	PI3K/Akt/mTOR	7,5
WZ8040	EGFR	Protein Tyrosine Kinase	7,5
XAV-939	Wnt/beta-catenin	Stem Cells & Wnt	7,5
XL019	JAK	JAK/STAT	7,5
XMD8-92	ERK	MAPK	7,5
Y-27632 2HCl	ROCK	Others	7,5
YM201636	PI3K	PI3K/Akt/mTOR	7,5
YO-01027	Gamma-secretase	Proteases	7,5
ZM 306416	VEGFR	Protein Tyrosine Kinase	7,5
ZM 323881 HCl	VEGFR	Protein Tyrosine Kinase	7,5
ZM 336372	Raf	MAPK	7,5
ZM 39923 HCl	JAK	JAK/STAT	7,5
ZM 447439	Aurora Kinase	Others	7,5
Zotarolimus(ABT-578)	mTOR	PI3K/Akt/mTOR	7,5
ZSTK474	PI3K	Neuronal Signaling	7,5
