# Supplementary material for: The drug-induced phenotypic landscape of colorectal cancer organoids
Source: Nat Commun. 2022 Jun 6;13:3135. doi: 10.1038/s41467-022-30722-9 (PMC9170716; doi:10.1038/s41467-022-30722-9)
Supplement: Supplementary file 2 — Reporting Summary [file 41467_2022_30722_MOESM2_ESM.pdf]

nature portfolio

Corresponding author(s): Michael Boutros and Matthias Ebert

Last updated by author(s): May 2, 2022

Reporting Summary

Nature Portfolio wishes to improve the reproducibility of the work that we publish. This form provides structure for consistency and transparency in reporting. For further information on Nature Portfolio policies, see our Editorial Policies and the Editorial Policy Checklist.

Statistics

For all statistical analyses, confirm that the following items are present in the figure legend, table legend, main text, or Methods section.

n/a	Confirmed	
☐	☒	The exact sample size (*n*) for each experimental group/condition, given as a discrete number and unit of measurement
☐	☒	A statement on whether measurements were taken from distinct samples or whether the same sample was measured repeatedly
☐	☒	The statistical test(s) used AND whether they are one- or two-sided<br>*Only common tests should be described solely by name; describe more complex techniques in the Methods section.*
☐	☒	A description of all covariates tested
☐	☒	A description of any assumptions or corrections, such as tests of normality and adjustment for multiple comparisons
☐	☒	A full description of the statistical parameters including central tendency (e.g. means) or other basic estimates (e.g. regression coefficient) AND variation (e.g. standard deviation) or associated estimates of uncertainty (e.g. confidence intervals)
☐	☒	For null hypothesis testing, the test statistic (e.g. *F*, *t*, *r*) with confidence intervals, effect sizes, degrees of freedom and *P* value noted<br>*Give P values as exact values whenever suitable.*
☒	☐	For Bayesian analysis, information on the choice of priors and Markov chain Monte Carlo settings
☐	☒	For hierarchical and complex designs, identification of the appropriate level for tests and full reporting of outcomes
☐	☒	Estimates of effect sizes (e.g. Cohen's *d*, Pearson's *r*), indicating how they were calculated

Our web collection on statistics for biologists contains articles on many of the points above.

Software and code

Policy information about availability of computer code

Data collection	High-throughput fluorescence Images were acquired with IN Cell Analyzer 6000 Aquisition Software v6.2, Lunienscence viability data was collected with MicroWin 2010 v. 5.24 software with Mithras readers. Microarrays were scanned using the Affymetrix GeneChip® Scanner 3000 with Affymetrix® GeneChip Command Console (AGCC) V4. QPCR data were collected with qTOWER qPCRsof (Analytik Jena) v3.4.6.0. Western blots were scanned with Fusion FX software (Vilber).
Data analysis	All software for organoid image analysis (including projection, segmentation, feature extraction, analysis of drug-induced phenotypes, live-dead-classification), all scripts for analysis of luminescence data, dose response relationships, expression, amplicon-seqencing and multi-omics factor analysis are available at: https://github.com/boutroslab/Supp_BetgeRindtorff_2021. This repository also contains a sample of the data to reproduce the figures.<br>Amplicon sequencing data were analyzed with BWA v.0.7.16, GATK v.3.8, ensembl-vep v.100.2 (ensembl v.91), FastQC v.0.11.5, TrimGalore v.0.4.3, Samtools v.1.3.1 and Picard v.1.138. Images were processed with Python v. 3.8.10 and R v.3.6.0 with the packages EBImage v. 4.20.0, MaxContrastProjection v. 1.6.1 and SCOPEAnalysis v.0.1.0. Gene expression, drug induced phenotypes and multi-omics analyses were performed with R v. 4.0.0, with affy v.1.62.0, limma v. 3.40.6, fgsea v.1.16.0, CMSclassifier v.1.0.0, scikit-learn v1.0, PharmacoGx v.1.8.3, Clusterprofiler v.4.2, and MOFA2 v.1.0.1 packages.

For manuscripts utilizing custom algorithms or software that are central to the research but not yet described in published literature, software must be made available to editors and reviewers. We strongly encourage code deposition in a community repository (e.g. GitHub). See the Nature Portfolio guidelines for submitting code & software for further information.

Data

Policy information about availability of data

All manuscripts must include a data availability statement. This statement should provide the following information, where applicable:

- Accession codes, unique identifiers, or web links for publicly available datasets
- A description of any restrictions on data availability
- For clinical datasets or third party data, please ensure that the statement adheres to our policy

Microarray data are available in Gene Expression Omnibus (GEO) under accession no. GSE117548 [https://www.ncbi.nlm.nih.gov/geo/query/acc.cgi?acc=GSE117548] and under https://github.com/boutroslab/Supp_BetgeRindtorff_2021/tree/master/data/. Raw Amplicon sequencing data are available through controlled access in the European Genome Phenome Archive (EGA, https://www.ebi.ac.uk/ega/home, accession no. EGAD00001004313, https://ega-archive.org/datasets/EGAD00001004313) to adhere with donating patient's data security and informed consent. Data access requests for sequence data will be evaluated and transferred within EU upon completion of a data transfer agreement and authorization by the data access committee of Division Signaling and Functional Genomics, DKFZ and Department of Medicine II, Medical Faculty Mannheim upon reasonable request to j.betge@dkfz.de or the corresponding authors. Processed sequencing results are available in Supplementary Table S2. Imaging data can be made available upon request to the corresponding authors after completion of a data transfer agreement and under the premise of adhering to EU General Data Protection Regulation. Processed, PCA-transformed feature data and all other data to reproduce the analyses for the figures are available under https://github.com/boutroslab/Supp_BetgeRindtorff_2021/. Source data are available in the Source Data File if applicable.

Field-specific reporting

Please select the one below that is the best fit for your research. If you are not sure, read the appropriate sections before making your selection.

☒ Life sciences ☐ Behavioural & social sciences ☐ Ecological, evolutionary & environmental sciences

For a reference copy of the document with all sections, see nature.com/documents/nr-reporting-summary-flat.pdf

Life sciences study design

All studies must disclose on these points even when the disclosure is negative.

Sample size	Organoids from 13 patients with colorectal cancer were extracted for this study. A total of 5.5 million organoids were analyzed by image based phenotyping.
Data exclusions	Data from two PDO lines (D015T and D021T) screened had to be excluded from image analysis due to too many out of focus objects.
Replication	For all experiments, at least two independent biological replicates were performed. For high-throughut profiling, two independen biological experiment were performed with each profiled organoid line. For all other experiments, the number of replicates is presented in each figure legend.
Randomization	Randomization was not relevant as samples were not allocated into experimental groups.
Blinding	Blinding was not relevant, since the automatized analysis of our data avoided subconcious manipulation due to any preconceived expectations concerning its outcome.

Reporting for specific materials, systems and methods

We require information from authors about some types of materials, experimental systems and methods used in many studies. Here, indicate whether each material, system or method listed is relevant to your study. If you are not sure if a list item applies to your research, read the appropriate section before selecting a response.

Materials & experimental systems

n/a	Involved in the study	
☐	☒	Antibodies
☐	☒	Eukaryotic cell lines
☒	☐	Palaeontology and archaeology
☒	☐	Animals and other organisms
☐	☒	Human research participants
☒	☐	Clinical data
☒	☐	Dual use research of concern

Methods

n/a	Involved in the study	
☒	☐	ChIP-seq
☒	☐	Flow cytometry
☒	☐	MRI-based neuroimaging

Antibodies

Antibodies used	IRS1 (06-248, Merck-Mllipore/Sigma-Aldrich), HSP-90 (sc-13119, Santa Cruz).
Validation	All antibodies were obtained from commercial source and used according to manufacturers instructions. IRS1 06-248 is a commonly used antibody and has been validated by co-IP and knockdown in several publications, for instance Drakas et al., PNAS 2004, or Yoneyama et al., eLife 2018, please also compare: https://scicrunch.org/resolver/AB_2127890. HSP-90 antibody has been used and validated in several publications, for instance by knockdown, overexpression and IP in Kuan et al. J. Biol Chem 2017.

Eukaryotic cell lines

Policy information about cell lines

Cell line source(s)	Patient derived organoids were derived from 13 patients with colorectal cancer at University Medical Center Mannheim, Heidelberg Univeristy.
Authentication	No authentication was possible as no common cell lines were used
Mycoplasma contamination	Absence of mycoplasma infection was confirmed by regular testing.
Commonly misidentified lines (See ICLAC register)	No commonly misidentified cell lines were used in the study.

Human research participants

Policy information about studies involving human research participants

Population characteristics	We extracted PDOs from a total of 13 patients with colorectal cancer, 5 of them female, 8 male, with a mean age of 66 years. 7 patients had a primary rectum carcinoma, 6 a primary colon carcinoma. Further details are provided in supplementary table S1.
Recruitment	All patients were recruited at University Medical Center Mannheim, Heidelberg University, Mannheim, Germany. We included untreated patients with a new diagnosis of colon or rectal cancer in this study and obtained biopsies from their primary tumors via endoscopy or from metastases via sonography-guided biopsy. Exclusion criteria were active HIV, HBV or HCV infections. Clinical data, tumor characteristics and molecular tumor data were pseudonymized and collected in a database. Selection bias was limited as much as possible, however, the study population was recruited at a single center, enriching the population for caucasians.
Ethics oversight	The study was approved by the Medical Ethics Committee II of the Medical Faculty Mannheim, Heidelberg University (Reference no. 2014-633N-MA and 2016-607N-MA). All patients gave written informed consent before tumor biopsy was performed.

Note that full information on the approval of the study protocol must also be provided in the manuscript.
